# Varied Effects of Carnosine and an Antioxidant-Enriched Carnosine Supplement in Paracetamol-Induced Hepatotoxicity Model in Mice

**DOI:** 10.3390/cimb48060581

**Published:** 2026-06-01

**Authors:** Dragana Zaklan, Nikola Martić, Bojana Andrejić Višnjić, Milana Bosanac, Snežana Hadžistević, Aleksandar Rašković, Nebojša Pavlović

**Affiliations:** 1 Department of Pharmacy, Faculty of Medicine, University of Novi Sad, 21000 Novi Sad, Serbia; dragana.zaklan@mf.uns.ac.rs; 2 Department of Pharmacology, Toxicology and Clinical Pharmacology, Faculty of Medicine, University of Novi Sad, 21000 Novi Sad, Serbia; nikola.martic@mf.uns.ac.rs (N.M.); aleksandar.raskovic@mf.uns.ac.rs (A.R.); 3 Department of Histology and Embryology, Faculty of Medicine, University of Novi Sad, 21000 Novi Sad, Serbia; bojana.andrejic-visnjic@mf.uns.ac.rs (B.A.V.); milana.bosanac@mf.uns.ac.rs (M.B.); 4 Institute of Pharmacology and Toxicology, Faculty of Medicine, University of Pristina in Kosovska Mitrovica, 38220 Kosovska Mitrovica, Serbia; s.hadzistevic@med.pr.ac.rs

**Keywords:** carnosine, hepatotoxicity, dietary supplements, CYP2E1, lipid peroxidation

## Abstract

Carnosine is widely recognized for its antioxidant and cytoprotective properties and is being increasingly used in dietary supplements. However, its effects in drug-induced liver injury remain insufficiently studied. This study aimed to investigate and compare the hepatoprotective and antioxidative effects of pure carnosine and an antioxidant-enriched commercial carnosine supplement in a murine model of paracetamol-induced hepatotoxicity. Adult male Swiss Webster mice were pretreated orally for seven days with carnosine or a commercial carnosine supplement prior to a single hepatotoxic dose of paracetamol. The serum biochemical parameters, hepatic oxidative stress markers, histopathology, and immunohistochemical expression of CYP2E1, COX-2, and Iba1 were evaluated 24 h after paracetamol administration. Paracetamol increased serum aminotransferases, lipid peroxidation, CYP2E1 expression, and histological liver injury. Pure carnosine pretreatment tended to exacerbate biochemical liver injury, whereas the commercial supplement attenuated lipid peroxidation, preserved bilirubin levels, and reduced histological damage. Both formulations decreased CYP2E1 expression and were associated with less necrosis and COX-2 immunoreactivity compared with paracetamol alone. Antioxidant enzyme activities and macrophage markers showed no consistent intergroup differences. These findings indicate that carnosine may not consistently exert hepatoprotective effects in acute drug-induced liver injury and that accompanying antioxidants may critically modify its biological actions.

## 1. Introduction

The global prevalence of noncommunicable diseases and lifestyle-related health problems burdening the increasingly aging world’s population, the shift from curative to preventive health care strategies, and the growing awareness and interest in wellness in modern societies have secured the steady growth in worldwide sales of nutraceuticals and dietary supplements in recent decades [1,2]. Since the pandemic of the coronavirus disease 2019, the global dietary supplement market has generated even greater revenue, approximated at $161.2 billion in 2021 and projected at $321.2 billion in 2028. This expansion has also propelled the advancement of scientific research within the field and an increase in the number of products available on the market [3,4,5]. Although there is no global agreement on the regulatory definition of dietary supplements, they are usually referred to as a vast spectrum of products available in diverse pharmaceutical forms, containing one or more nutrients or other ingredients, intended to supplement the normal diet and provide beneficial nutritional or physiological effects [6]. Namely, the listed components may include minerals, vitamins, amino acids, enzymes, fatty acids, and various botanicals, among others [1,6].

The popularity of and the demand for dietary supplements is ever-growing, since consumers seek to improve or maintain their overall well-being and prevent or treat medical conditions. Previous studies have revealed that the prevalence of use ranges from 22% to 53% in developed countries. Furthermore, many consumers use more than one product for a prolonged time. Also, when opting for supplementation, fewer of them rely on advice from health care professionals, while many fail to report that they consume such products, unaware of the potentially detrimental health effects. This may be due to the common misconception that dietary supplements are safe to use and do not pose health risks, since they are obtained from natural sources [1,7,8]. Nevertheless, it is well documented that these products may cause a spectrum of (unexpected) adverse reactions. For instance, these may be the results of product contamination or adulteration. Interactions with prescription and over-the-counter (OTC) therapeutics, as well as other nutrients, should be carefully considered and not overlooked. An astonishing 80% of dietary supplements are estimated to likely interplay with cytochrome P450 enzymes [6]. Hepatotoxicity is gaining more recognition, as 2–16% of all cases of drug-induced liver injuries (DILIs) are considered to stem from dietary supplement consumption [9]. Interestingly, as revealed by a recent study, the prevalence of dietary supplement use for therapeutic purposes among patients suffering from liver disease was 14.6% [10]. A staggering 23,000 annual emergency department visits were associated with the use of these products in the US [11]. Accordingly, around 35% of pharmacists and physicians reported having encountered the adverse effects caused by dietary supplements. Nevertheless, several studies estimate that 82% to 98% of adverse events associated with dietary supplement use remain unreported by health care professionals, which is of particular concern [12,13]. Additionally, in a cross-sectional online survey, only one-third of respondents reported consulting a physician or a pharmacist before consuming dietary supplements [10], while another study conducted in a hospital setting found that only 20% of inpatients reported being asked about dietary supplement use by a physician or a nurse at admission [14]. Undoubtedly, adverse effects related to dietary supplement consumption are undetected, underreported, and insufficiently documented [15,16]. It is evident that science and legislation still lag behind the ever-expanding market, and a comprehensive and interdisciplinary approach to ensure quality, safety and efficacy of dietary supplements is greatly needed. In this sense, preclinical investigations of therapeutic and toxic potential of compounds are of immense importance [17].

β-Alanyl-L-histidine, widely known as carnosine, is a dipeptide typically present in vertebrates, and abundant in the tissues with the most active oxidative metabolism, including human skeletal muscle, the brain, the heart, and gastrointestinal tissues [18]. Since its discovery in 1900 by V.S. Gulewitch, carnosine has been sparking the interest of scientists determined to unveil the physiological roles and investigate the therapeutic potential of this biomolecule. More than a century later, while some of its properties have been clarified, many are yet to be elucidated in order to gain comprehensive insight into the role of carnosine in various physiological and pathophysiological conditions [19,20] In particular, antioxidative potential is one of the most commonly attributed properties to carnosine, since it acts in both direct and indirect manners, maintaining pH homeostasis, quenching and detoxifying radical and oxidizing species, thereby protecting the cellular machinery. The role of carnosine in protection from oxidative stress is mediated by its ability to chelate metal ions, scavenge reactive oxygen species (ROS) and peroxyl radicals, and detoxify hypochlorite (HOCl) and cytotoxic reactive carbonyl species (RCS), thus preventing the formation of advanced glycation and lipoxidation end products. Furthermore, a decrease in oxidative stress and inflammation may be attributed to its immunomodulatory properties [18,21]. For these reasons, a great effort is being made to utilize carnosine in the treatment of a vast spectrum of oxidative-stress-induced pathologies, including neurodegenerative, malignant, cardiometabolic, and ocular diseases, among others [22]. Similarly, extensive research is being directed towards the potentially protective effects of carnosine in oxidative liver damage [23]. However, further investigation is needed to fully characterize the multimodal nature and effects of carnosine, both beneficial and potentially detrimental, and exploit its therapeutic potential.

Therefore, in light of the rising need for dietary supplement safety and efficacy investigation, as well as carnosine’s therapeutic potential, the present study aimed to investigate the hepatoprotective and antioxidative potential of carnosine and a commercial carnosine supplement in paracetamol-induced hepatotoxicity in mice.

## 2. Materials and Methods

### 2.1. Materials

L-Carnosine was gifted by CarnoMed (Novi Sad, Serbia). A commercial dietary supplement of carnosine (Karnozin extra^®^, CarnoMed, Novi Sad, Serbia, batch No. C66S110719) that contains L-carnosine (125 mg), coenzyme Q10 (20 mg), L-carnitine (20 mg), vitamin E (20 mg), and standardized grape seed (20 mg) and north blueberry seed extract (20 mg) in each capsule was used as well. Paracetamol was acquired from Sigma-Aldrich, St. Louis, MO, USA. All the chemicals and commercial kits applied for the biochemical and immunohistochemical analyses were used as received without any further modifications.

### 2.2. Experimental Design and Animal Treatment

The study was conducted on healthy adult Swiss Webster male laboratory mice, weighing 25–30 g. The animals, obtained from the Military Medical Academy (Belgrade, Serbia), were maintained in the vivarium of the Institute of Pharmacology, Toxicology and Clinical Pharmacology at the Faculty of Medicine, University of Novi Sad, in Ehret Uni-Protect cabinets equipped with a high-efficiency particulate air (HEPA) filter (EHRET Labor und Pharmatechnik GmbH&Co. KG, Emmendingen, Germany). The animals were kept under standard laboratory conditions in polycarbonate transparent cages, under a 12 h day/night circadian cycle, at a controlled ambient temperature (22–25 °C) and air humidity (55 ± 1.5%). Throughout the experiment, all mice were allowed access to standard pellet food and tap water ad libitum. At the end of the seven-day treatment, the animals were fasted for 12 h before and 6 h after receiving a single toxic dose of paracetamol (110 mg/kg, p.o.), while water was provided ad libitum. The animal care and experimental procedures adhered to ethical guidelines provided by the EU Directive 2010/63/EU on animal welfare and the Law of Animal Welfare of the Republic of Serbia (OG RS 41/09). The study was approved by the Ethical Commission for the Protection of Animal Welfare of the University of Novi Sad (Novi Sad, Serbia; No. 01-107/6-1) and the Veterinary Administration of the Ministry of Agriculture, Forestry and Water Management of the Republic of Serbia (Belgrade, Serbia; No. 23-07-04785/2019-05).

A total of 36 mice were randomly divided into 6 experimental groups, each containing six individuals. The groups were distributed as follows:

- Con—control group, saline solution at 1 mL/kg p.o.
- ConP—saline solution at 1 mL/kg p.o. + single dose of paracetamol at 110 mg/kg p.o.
- Car—carnosine at 370 mg/kg p.o.
- Car+P—carnosine at 370 mg/kg p.o. + single dose of paracetamol at 110 mg/kg p.o.
- CarS—carnosine supplement at 666 mg/kg p.o.
- CarS+P—carnosine supplement at 666 mg/kg p.o. + single dose of paracetamol at 110 mg/kg p.o.

The doses of the tested compounds were adapted to mice and calculated using the formula for converting between human and animal doses, from the standard human dose. The animals were treated orally by gavage at the same time each day, over a seven-day period, by a trained individual. Both the carnosine and the carnosine supplement were stored at room temperature and freshly dispersed in saline solution every day, 30 min before the treatment. Paracetamol was stored at room temperature and dissolved in saline solution at 55 °C using a magnetic stirrer, then cooled to 37 °C immediately before administration. Sacrifices were performed by decapitation 24 h after a single toxic dose of paracetamol was administrated. Blood and liver tissues were sampled for further analysis.

### 2.3. Serum Biochemical Parameter Analysis

Serum samples were used to determine the enzymatic activity of aspartate aminotransferase (AST, EC 2.6.1.1) and alanine aminotransferase (ALT, EC 2.6.1.2) and the concentrations of total and direct bilirubin, as an indicator of the extent of hepatocellular damage. To evaluate the renal excretory function, the concentrations of urea, creatinine and uric acid were measured. Biochemical analyses were performed using well-established spectrophotometric methods utilizing commercially available kits, in accordance with the provided instruction manuals and automatic analyzer AU480 (Beckman Coulter Inc., Indianapolis, IN, USA).

### 2.4. In Vivo Antioxidant Activity

Liver homogenates were prepared using obtained liver tissues and TRIS-HCl-buffered solution in a ratio of 1:3 (*w*/*v*) at 4 °C, utilizing a Potter homogenizer. Lipid peroxidation (LP) and oxidative stress parameters were quantified using the aforementioned liver homogenates, in duplicate for each sample. The LP intensity was evaluated indirectly by determining the quantity of malondialdehyde (MDA), as a byproduct of lipid breakdown. The activities of catalase (CAT), glutathione peroxidase (GPx), glutathione reductase (GR), and glutathione-S-transferase (GST) were also determined by utilizing previously published spectrophotometric methods [24,25].

### 2.5. Histopathology and Immunohistochemistry Assessment

Liver tissue samples were fixed by immersion in 4% paraformaldehyde (for 24 h) and dehydrated in graded ethanol solutions (70%, 80%, 96%, and absolute), cleared in 100% xylene, and infiltrated and embedded in paraffin blocks on a casting console (Sakura Finetek, Chuo-ku, Tokyo, Japan). The tissue was then cut into 5 µm slices on a microtome (Sakura Finetek, Chuo-ku, Tokyo, Japan) and transferred to a water bath (DiaPath, Martinengo, Italy), after which the slices were transferred to a glass slide and a thermostat (Memmert GmbH Co. KG, Büchenbach, Germany). The tissue was stained with standard hematoxylin and eosin (HE) and periodic acid–Schiff (PAS) staining for a qualitative assessment (presence of necrosis, inflammatory infiltrate, hepatocyte changes, etc.).

In addition to standard (H&E) staining, immunohistochemical staining was performed. After rehydration, formalin-fixed, paraffin-embedded liver sections were subjected to heat-induced retrieval in citrate buffer (pH of 6.0), heated for 25 min at a pressure of 70–80 Pa and subsequently allowed to cool at room temperature for 20 min. After cooling, the sections were washed in EnVision™ FLEX Wash Buffer (1:20, Agilent, Santa Clara, CA, USA). Endogenous peroxidase activity was blocked by incubation with EnVision™ FLEX peroxidase-blocking reagent (ready-to-use) for 12 min at room temperature. The sections were then washed and incubated with primary antibodies against cyclooxygenase-2 (COX-2; 1:500, ab283574, Abcam, Cambridge, UK), cytochrome P450 2E1 (CYP2E1; 1:200, CSB-PA006425EA01H4, Flarebio, College Park, MD, USA), or ionized calcium-binding adapter molecule 1 (Iba1; 1:8000, ab178847, Abcam, Cambridge, UK).

Following primary antibody incubation, the sections were washed in EnVision™ FLEX wash buffer and incubated for 20 min with an HRP-conjugated secondary antibody using the EnVision™ FLEX detection system (ready-to-use). Immunoreactivity was visualized with EnVision™ FLEX DAB+ chromogen, prepared by mixing 1 mL of EnVision™ FLEX substrate buffer with one drop of EnVision™ FLEX DAB+ chromogen and applying for 10 min.

The sections were counterstained with hematoxylin for 10 min, dehydrated, cleared, and mounted using DPX mounting medium (Sigma-Aldrich Chemie GmbH, Darmstadt, Germany).

For the quantitative analysis of the immunohistochemically stained liver sections, the tissue was photographed using a Leica DM LB light microscope equipped with a Leica DC100 digital camera. The acquired images were analyzed and processed using the Leica Application Suite software. For the quantitative analysis, the ImageJ software (version 1.53; National Institutes of Health, Bethesda, MD, USA; freely available at https://imagej.net/software/fiji/downloads, accessed 18 January 2019) was used.

In the liver sections immunostained with anti-Iba1 antibodies, five randomly selected fields at 200× magnification were photographed. The images were analyzed using the ImageJ software to determine the number of Iba1-positive and Iba1-negative cells, using the Plugins > Analyze > Cell Counter tool.

In the sections immunostained with anti-CYP2E1 antibodies, five randomly selected fields per animal were photographed. In ImageJ, the image contrast was enhanced using the Process > Enhance Contrast command with an intensity of 3%. CYP2E1-positive pixels were selected based on the staining intensity using the Color Threshold tool. The area of the selected pixels was measured, and the resulting data were further processed using the Microsoft Office Excel 2019 software.

COX-2 immunoexpression was assessed descriptively because the focal, heterogeneous, and predominantly perinecrotic distribution pattern made automated area-based threshold quantification unreliable in randomly sampled microscopic fields.

### 2.6. Statistical Analysis

The data are expressed as the mean ± standard deviation (SD). Normality of the distribution was assessed using the Shapiro–Wilk test. For data not conforming to a normal distribution, a comparison was performed using the Kruskal–Wallis nonparametric test, followed by Dunn’s post hoc test. For normally distributed data, a one-way analysis of variance (ANOVA) followed by Tukey’s multiple comparison test was applied. Differences were considered significant when *p* < 0.05. The data were analyzed using the Origin 2018 software (OriginLab Corp, Northampton, MA, USA).

## 3. Results and Discussion

As previously mentioned, there is a growing interest in nutraceuticals and dietary supplements from both the scientific community and the general public, as a means of improving health and well-being. Meanwhile, drug expenditures and their growth rate have drawn the attention of health policymakers, as a wide range of therapeutic options have been employed to address multimorbidity in an increasingly aging society [26,27]. Consequently, DILIs have emerged as a significant public health concern, characterized by unpredictable and potentially severe adverse reactions to medications and dietary supplements, as the liver is the primary organ responsible for xenobiotic metabolism and elimination. According to the previous reports, drug hepatotoxicity affects 1–1.5 million people globally [28]. Paracetamol, also known as acetaminophen, is the most frequently used analgesic and antipyretic drug worldwide, generally well tolerated by adults and children and present in numerous prescription and OTC medicines. Due to its wide availability, intentional or accidental overdose may occur (over 4 g/day), which can lead to a hospital admission due to dose-dependent hepatotoxicity, acute liver failure and renal damage [29,30,31]. Moreover, under certain conditions, such as chronic alcohol use or co-administration of other drugs, paracetamol hepatotoxicity can occur even at therapeutic doses. Therefore, the search for clinically relevant antidotes, apart from well-established N-acetylcysteine, for paracetamol-induced hepatotoxicity has been ongoing for several decades. At the same time, paracetamol-induced liver injury in mice has been a widely used experimental model in preclinical research to evaluate compounds with hepatoprotective and antioxidative properties, owing to its high reproducibility, dose-dependent hepatotoxicity and translational importance [29,31,32]. Hence, this study aimed to investigate the effects of carnosine and a commercial carnosine dietary supplement treatment on the liver and kidney function, as well as the oxidative status, in mice with paracetamol-induced hepatotoxicity.

The pathophysiology of liver injury induced by administration of a toxic dose of paracetamol in mice closely resembles that observed in human hepatocytes, with a difference in time course of the injury. Furthermore, previous studies have shown that male mice exhibit a greater susceptibility to paracetamol overdose. Therefore, according to a previously established model, we administered 110 mg/kg of paracetamol to sexually mature male laboratory mice [25,33]. The major mechanism of the drug’s hepatotoxicity is its metabolic activation in the liver by cytochrome P450 (primarily CYP2E1, CYP1A2 and CYP3A4), which generates N-acetyl-p-benzoquinone imine (NAPQI) as a reactive metabolite. Once the NAPQI production exceeds the hepatic glutathione (GSH) detoxification capacity, NAPQI covalently binds to various cell proteins, causing oxidative stress, lipid peroxidation, excessive generation of free radicals, metabolic alterations, endoplasmic reticulum stress, extensive mitochondrial damage and DNA fragmentation, which leads to cell necrosis [28,29,31,34]. As presented in Table 1, administration of a single toxic dose of paracetamol caused an increase in serum ALT activity, compared to a control group. Treatment with carnosine or a commercial carnosine dietary supplement alone did not affect the ALT serum activity relative to the control. However, carnosine decreased the AST serum activity relative to the control, and appeared to potentiate paracetamol toxicity, as indicated by the ALT and AST values in the pretreated group. Furthermore, the carnosine dietary supplement did not ameliorate paracetamol-induced liver injury, but appeared to exert less dramatic effects on the AST levels compared to pure carnosine. ALT remains one of the most widely used biomarkers for hepatocyte injury, and elevated activities of aminotransferases are characteristic of the hepatocellular damage caused by paracetamol overdose [26,35]. Although no statistically significant differences were observed, the increased concentrations of total and direct bilirubin in the group treated with carnosine for 7 days prior to a single toxic dose of paracetamol suggest that this combination may possess hepatotoxic potential. Conversely, the commercial carnosine dietary supplement did not cause as evident of an increase in bilirubin levels, and even maintained direct bilirubin concentrations comparable to those of the control. These results conflict with previous findings. Yan et al. reported that the pretreatments of carnosine dose-dependently alleviated paracetamol-induced elevation of ALT and AST in plasma of Balb/cA mice. However, carnosine was supplemented through drinking water (0.5–2 g/L) and the pretreatment lasted for 4 weeks prior to paracetamol administration (350 mg/kg) [36]. The carnosine treatment also reduced the serum ALT, AST and γ-glutamyl transpeptidase (GGT) in male Sprague Dawley rats with CCl_4_-induced hepatotoxicity, as well as reduced the total bilirubin to the normal levels of the control [37]. Similar protective effects were reported for female Wistar rats with thioacetamide-induced liver injury [38], male albino mice with cadmium-induced liver injury [39] and binge-ethanol-administered female Wistar rats [40]. Furthermore, carnosine, alone and in combination with vitamin E, decreased the serum ALT and AST activities in male doxorubicin-treated Sprague Dawley rats, ameliorating its hepatotoxic effects [41]. Neither carnosine nor the commercial dietary supplement used in this study, alone or in combination with simvastatin, altered the serum transaminase levels in high-fat-diet-fed adult male Wistar rats [42]. Hepatotoxic effects of certain compounds with previously reported hepatoprotective potential have been reported in the literature [43]. For instance, therapeutic doses of green tea extract provoked liver damage and even exacerbated paracetamol-induced hepatotoxicity in male albino rats, observed by augmented concentrations of liver enzymes and related biochemical and histopathological alterations indicating hepatocellular damage. The proposed mechanisms underlying such an outcome included oxidative stress and caspase 3-dependent apoptosis [44]. Pretreatment of male C57BL/6 mice with St. John’s wort (SJW) also exacerbated paracetamol-induced liver injury, as evidenced by a marked increase in liver architecture damage and extensive hepatocellular necrosis. Serum transaminase activities were markedly increased in the pretreated group compared to the group treated with paracetamol alone. The authors proposed that SJW potentiated the generation of toxic metabolites associated with CYP-mediated paracetamol bioactivation [45]. Conflicting results have been reported for ω-3 fatty acids as well. Even though previous studies have reported protective effects of fish oil in other models of liver diseases, in the case of paracetamol intoxication, it appeared to potentiate the drug’s toxicity by elevating the serum ALT and the extent of hepatocellular necrosis in male C57BL6/J mice [46]. Yifan et al. reported the paradoxical toxigenic transformation of picroside II, a natural antioxidant, which further aggravated liver injury in paracetamol-, CCl_4_- and D-galactosamine-induced hepatotoxicity in rats [47]. Interestingly, although it is generally considered that bilirubin acts as an antioxidant through the biliverdin–bilirubin redox cycle, recent studies have reported conflicting findings on its reputedly protective effects. This controversy may be associated with the activity of nuclear factor erythroid 2-related factor 2 (Nrf2), a critical regulator of cellular antioxidant defense mechanisms that orchestrates a complex antioxidant cellular machinery. Wang et al. reported that activation of Nrf2 increased the expression of heme oxygenase-1 (HO-1), which resulted in detrimental effects in a cholestatic liver injury model. HO-1 degrades heme to biliverdin, which is subsequently converted to bilirubin, along with the generation of carbon monoxide and free iron. The authors reported that activation of the Nrf2/HO-1 pathway led to excessive accumulation of bilirubin and impaired mitochondrial function, while an Nrf2 knockout conferred hepatoprotection in mice with cholestatic liver injury [48]. Since carnosine has been found to enhance the expression and activation of the Nrf2 pathway, as discussed later in the manuscript, it may be hypothesized that this mechanism, along with oxidative stress, could contribute to the elevated bilirubin levels in animals treated with pure carnosine prior to paracetamol administration. This phenomenon was not observed in animals pretreated with the commercial dietary supplement, which may be attributed to the presence of its additional constituents, as discussed later in the manuscript.

Serum concentrations of urea, creatinine, and uric acid were also measured as markers of renal function. The results are presented in Table 2. The applied toxic single dose of paracetamol did not significantly alter the concentrations of urea or creatinine compared to the control. This is consistent with literature reports showing that substantially higher doses of paracetamol (300–750 mg/kg) are needed to induce severe nephrotoxicity [49,50,51]. However, a decrease in the concentration of uric acid was evident, although statistical significance was not reached. Neither carnosine nor its commercially available dietary supplement alone caused a disturbance in renal function compared to the control. Notably, carnosine treatment prior to paracetamol administration led to an even greater decrease in the uric acid concentration compared to paracetamol alone, although statistical significance was not reached. Since uric acid serves as an endogenous antioxidant, these results indicate heightened oxidative stress in the animals that received the carnosine pre-treatment. Our findings are not in accordance with previous reports. The literature data regarding nephroprotective effects suggest that a carnosine pre-treatment may normalize serum urea and creatinine levels and attenuate histopathological markers of kidney damage in rat models of renal injury induced by nephrotoxic agents such as gentamicin [52], ifosfamide [53] and nickel [54].

**Figure 1:**
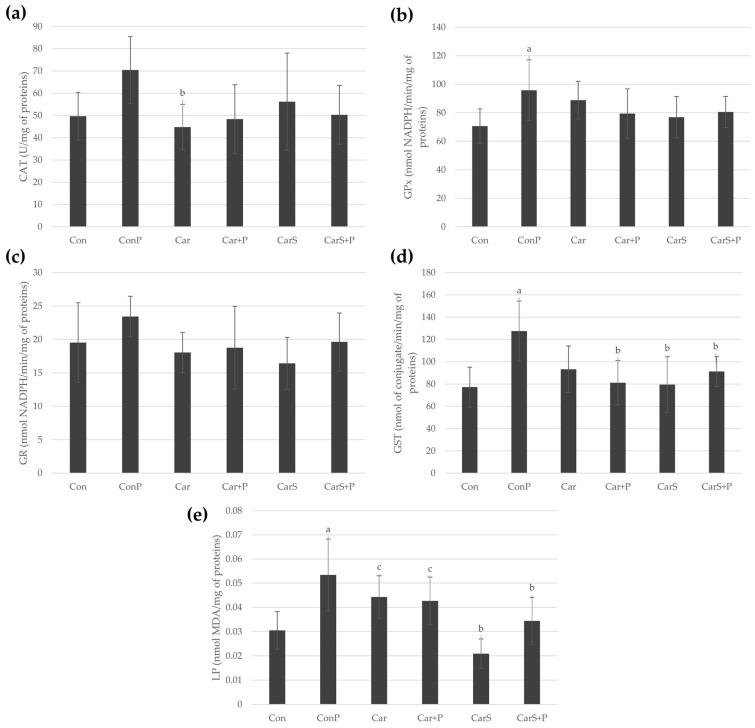
Activities of (**a**) catalase (CAT), (**b**) glutathione peroxidase (GPx), (**c**) glutathione reductase (GR), (**d**) glutathione-S-transferase (GST) and (**e**) lipid peroxidation intensity (LP) in liver homogenates, according to animal treatment. ^a^ Significantly different from Con group, *p* < 0.05; ^b^ significantly different from ConP group, *p* < 0.05; ^c^ significantly different from CarS group, *p* < 0.05.

As previously mentioned, in the case of paracetamol overdose, excessive NAPQI reacts with available GSH, depleting its cytoplasm storage in both the cytosol and mitochondria. After GSH is exhausted, NAPQI is free to react with alternative targets, setting in motion a series of events that lead to hepatic injury and, ultimately, cell necrosis. One of these is mitochondrial dysfunction occurring as a consequence of NAPQI binding to mitochondrial proteins, which is associated with ROS generation, inhibition of mitochondrial respiration and a decrease in ATP levels [26]. Induction of oxidative stress is widely reported as a manifestation of paracetamol hepatotoxicity. Initial oxidative stress is responsible for the activation of the mitogen-activated protein kinase cascade resulting in the phosphorylation of c-Jun N-terminal kinase (P-JNK) in the cytosol, ultimately resulting in the impairment of the mitochondrial electron transport chain and further amplification of the initial oxidative/nitrosative stress. Electron leakage leads to the formation of superoxide radicals, which are later converted to hydrogen peroxide (H_2_O_2_) or peroxynitrite (ONOO^−^). The endogenous antioxidant defense system, including GSH, CAT, GP_x_, GR, and GST, is employed to attenuate potential oxidative damage. Otherwise, lipid peroxidation may occur, further damaging the cell structure and causing hepatocyte damage and necrosis [28,31,55]. The role of dietary and nutraceutical antioxidants in mitigating paracetamol-induced liver injury has been widely discussed [26]. To evaluate the antioxidative potential of carnosine and its commercial dietary supplement, LP was measured through the MDA level, while the activities of CAT, GP_x_, GR and GST were also determined. The corresponding results are presented in Figure 1. As expected, the MDA concentration was significantly elevated in the paracetamol-treated group relative to the control, indicating enhanced LP. Neither carnosine nor the commercially available formulation alone caused an increase in the LP intensity or disturbance of the oxidative status compared to the control. The commercial carnosine dietary supplement prevented the increase in MDA levels exposed to a single toxic dose of paracetamol, suggesting a potentially protective effect against paracetamol-induced LP. The same treatment was also more successful in decreasing the MDA levels compared with pure carnosine. LP is a free-radical reaction process that is still regarded as one of the mechanisms of cell death, more recently described as ferroptosis, caused by paracetamol hepatotoxicity. The presence of ROS leads to the oxidation of fatty acids, especially polyunsaturated fatty acids (PUFAs), in lipid membranes, leading to membrane breakdown and, ultimately, cell necrosis. MDA is the most abundant aldehyde product resulting from LP, and thus represents a valuable biomarker of oxidative-stress-related paracetamol toxicity [30,55]. The activities of other enzymes indicative of oxidative stress in liver homogenates support the presence of antioxidant potential in both carnosine formulations. Notably, pre-treatment with the commercial formulation led to a statistically significant decrease in the GST concentration compared to the group exposed to the toxic single dose of paracetamol. This may indicate that carnosine acts synergistically with additional antioxidant constituents present in the commercial dietary supplement to reduce a demand for GST-mediated detoxification. Our findings are partly consistent with previously published studies. Both direct and indirect antioxidant activity of carnosine have been reported in in vitro and in vivo studies. Carnosine exerts its direct antioxidant activity, acting as a non-enzymatic free-radical scavenger, by interacting with products of LP, neutralizing toxic heavy metals and decreasing the intracellular levels of ROS and reactive nitrogen species (RNS), and ultimately reducing the extent of processes leading to the formation of advanced glycation and lipoxidation end products (AGEs and ALEs). Namely, its effective scavenging activity against molecules of a diverse nature, ranging from glucose to highly toxic carbonyl species, has been attributed to its imidazole moiety. The indirect antioxidant activity of carnosine is related to its ability to enhance the expression and activation of the Nrf2 pathway, which involves activation of the phosphatidylinositol-3-kinase (PI3K)/AKT signaling pathway. This cascade ultimately regulates the transcription of more than two hundred genes that contain an antioxidant response element (ARE) in their promotor region, including thioredoxin 1, superoxide dismutase-1, and catalase [21,56,57]. Several in vivo studies have reported the antioxidative potential of carnosine in various animal models of liver injury, including paracetamol-, CCl_4_-, thioacetamide-, cadmium-, binge ethanol-, and doxorubicin-induced hepatotoxicity [36,37,38,39,40,41]. Co-administration of paracetamol and carnosine increased the activities of antioxidant enzymes and reduced LP in the spinal cord of male Wistar rats in a chronic constriction injury model [58]. Conversely, in thioacetamide-induced liver cirrhosis in rats, carnosine reduced the elevated MDA levels, but had no effect on decreased activities of GSH, SOD and GSH-Px [59]. In a rat model of isoproterenol-induced myocardial infarction, carnosine supplementation reduced the MDA and diene conjugate concentrations and increased GSH, SOD and GSH-Px in the heart tissue; however, it had no effect on the aforementioned oxidative stress parameters in the plasma and erythrocytes [60]. To summarize, conflicting results have also been reported in the literature. Our study shows that a single toxic dose of paracetamol intensified the LP process. This is consistent with the findings of Mladenović et al., who reported that LP plays an important role in the early phase of paracetamol-induced liver injury, as evidenced by increased liver and plasma MDA levels [35]. Although the carnosine pre-treatment appeared to reduce LP, no statistical significance was observed. However, the commercial dietary supplement alleviated LP and reduced the GST levels, which is partially consistent with the previously discussed literature. Of note, we observed no significant changes in the examined oxidative stress parameters among the groups. This discrepancy may be explained by the complexity of paracetamol toxicity and the multiple cell defense mechanisms involved, which overlap and activate at different timepoints. Differences in animal models and the methods applied to induce hepatotoxicity contribute to variability in the outcomes. Additionally, the duration of treatment and the selected experimental endpoint can significantly affect the observed intervention outcomes [35,61]. Mladenović et al. reported that LP and increased RNS production were associated with liver injury within first 6 h after paracetamol intoxication and persisted for the next 48 h. On the other hand, antioxidant capacity of hepatocytes undergoes phasic alteration. After the initial decrease in GSH levels resulting from NAPQI-induced inactivation of sulfhydryl groups, adaptive responses occur, resulting in a compensatory increase in the GSH levels [35]. Of note, LP products may also act as signaling mediators to induce an adaptive antioxidant response and enhance the cell defense capacity [62]. However, even if antioxidant enzyme activities are maintained at control levels, they may not be sufficient to counteract LP occurring at cellular membranes, and the process may continue even in the presence of carnosine. On the other hand, the carnosine dietary supplement examined in this study contains additional components that may mitigate LP through their own antioxidant properties, potentially producing synergistic effects. Hepatoprotective and antioxidant properties are attributed to L-carnitine, and its deficiency has been shown to aggravate paracetamol hepatotoxicity [63]. Hepatoprotective effects of coenzyme Q10 have also been reported in rodent models of liver injury [64,65]. Vitamin E attenuated the harmful effects of acute paracetamol administration on the liver, as demonstrated by measures of oxidative stress in pregnant female Wistar rats [66]. Polyphenol-rich plant extracts, such as those from grape seeds and blueberry seeds, are also widely associated with antioxidant activity [67].

**Figure 2:**
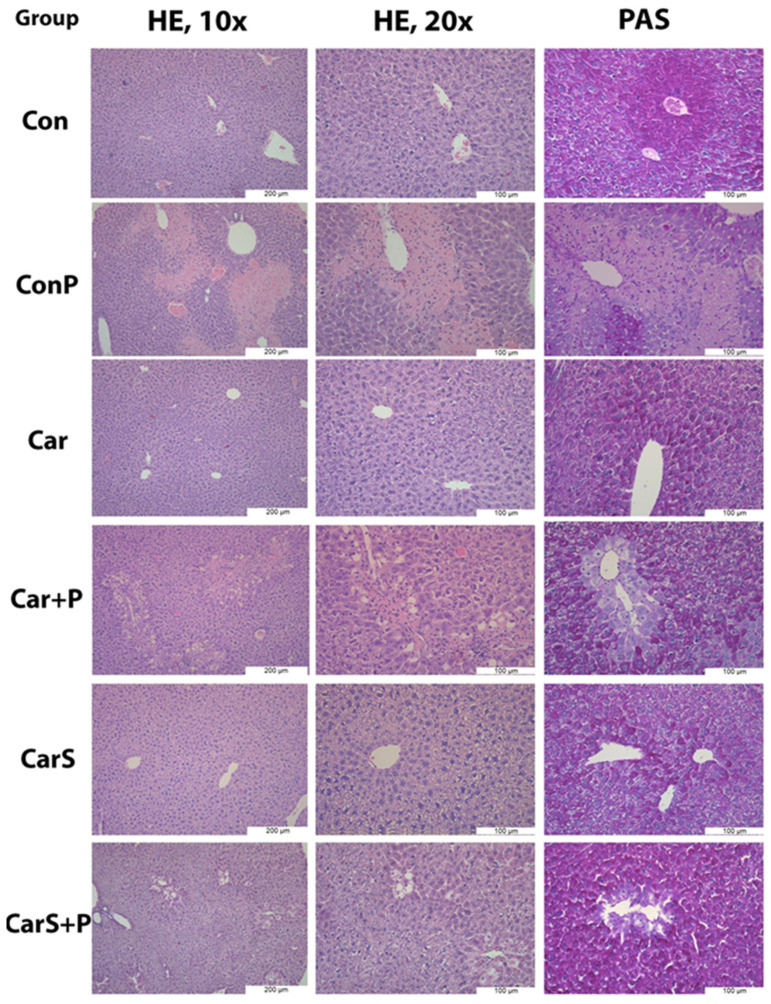
Liver tissue changes (HE and PAS staining, magnification of 10×, 20×).

As previously stated, the murine model of paracetamol overdose is a high-fidelity model with a broad translational relevance for human liver injury [29,31,32]. Paracetamol toxicity causes hepatocyte damage and produces a characteristic pattern of centrilobular necrosis (around the central vein, acinar zone 3), while in more severe cases, this can extend, causing massive, confluent necrosis [68]. Representative microphotographs of liver tissue and its alterations, stained with standard hematoxylin and eosin and the PAS stain, are shown in Figure 2. As expected, the control group (Con) exhibited a normal hepatic architecture. However, centrilobular necrosis was present in 60% of the ConP liver samples, along with hepatocyte vacuolization and degeneration, signs of apoptotic cell death (acidophilia of the cytoplasm; cariopicnosis) and lymphocytic inflammatory infiltrate. When present, the necrosis was confluent, almost bridging neighboring central parts of liver lobules. Centrilobular areas of the ConP livers exhibited glycogen depletion, visible in PAS staining. Neither carnosine alone (Car) nor the commercial carnosine dietary supplement (CarS) induced histological alterations. Coadministration of paracetamol and carnosine (Car+P group) revealed the presence of necrosis in 40% of subjects/animals. In samples where necrosis was present, it was centrilobular, but it affected much smaller areas of tissue compared to in the ConP group. The remaining 60% of the subjects/animals’ livers exhibited minor signs of hepatic degeneration or apoptosis, with no or minimal lymphocytic infiltrate. Necrosis was observed to a much lesser extent (rare, focal areas of a few affected cells) in the livers of all animals co-treated with a commercial carnosine dietary supplement and paracetamol (CarS+P group) compared to the ConP and Car+P groups. The observed occurrence and distribution of necrosis in the ConP group were expected and typical, while the less frequent presence of necrosis and the lesser involvement of liver tissue (Car+P and CarS+P) might suggest partial protection against paracetamol-induced toxicity by both carnosine formulations.

**Figure 3:**
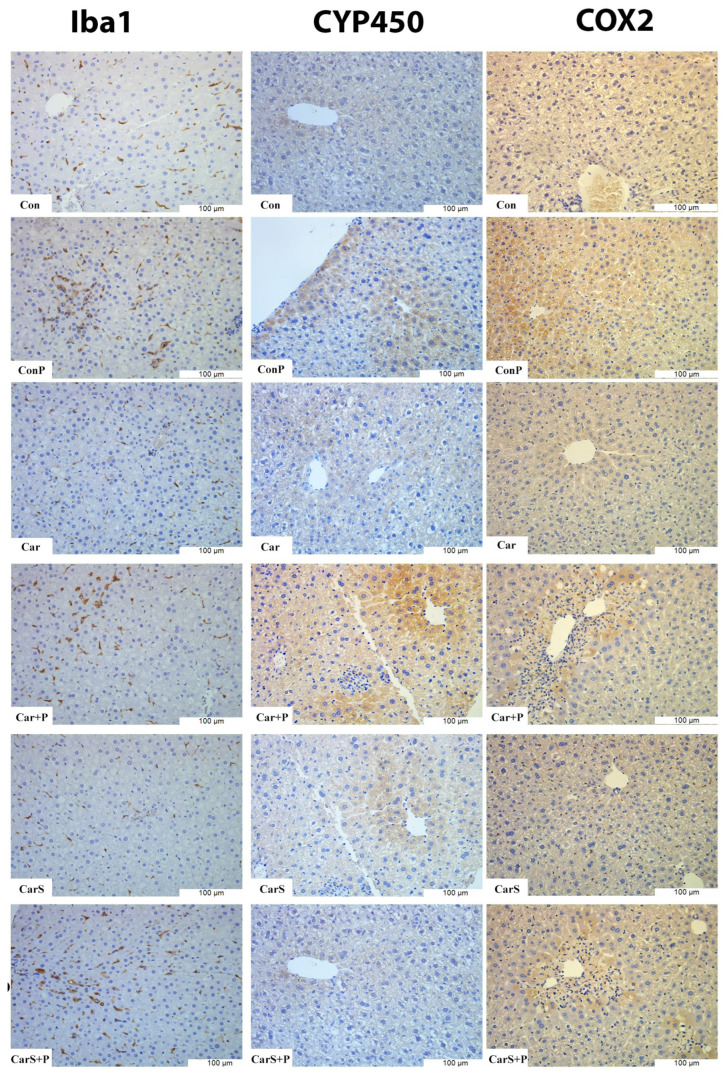
Expression of Iba1, CYP2E1 and COX2 in liver tissue (20×).

Therapeutic doses of paracetamol are primarily metabolized through glucuronidation and sulfation, and only a small fraction is metabolized by cytochrome P450 enzymes. On the other side, following an overdose, cytochrome P450 activity increases substantially, leading to excessive NAPQI formation [26]. Microphotographs of liver tissue immunohistochemically stained using the primary antibodies CYP2E1, Iba1 and COX2 are presented in Figure 3. A quantitative assessment of CYP2E1-positive liver tissue (CYP2E1^+^ surface area) revealed a significant increase following paracetamol treatment compared with the control group (Table 3), predominantly localized in centrilobular regions affected by necrosis and hepatocyte degeneration. Administration of carnosine or the commercial carnosine dietary supplement alone (Car and CarS groups) did not raise the CYP2E1 activity above the Con group level, as expected. Co-administration of paracetamol with either carnosine formulation maintained the CYP2E1 expression at the control levels and significantly reduced the CYP2E1 positivity relative to ConP. These findings suggest that both carnosine treatments attenuated paracetamol-induced CYP2E1 upregulation, suggesting a potential role in mitigating the associated oxidative stress.

The reduction in CYP2E1 expression in the Car+P and CarS+P groups suggests a certain level of excessive cytochrome P450 activity alleviation and NAPQI accumulation as a result of paracetamol toxicity, as well as resulting oxidant stress, mitochondrial matrix swelling and rupture of the outer mitochondrial membrane. These changes would lead to hepatocyte death, and it is noteworthy that necrosis was also alleviated or absent in those groups. Therefore, the ability of carnosine to affect cytochrome P450 is noteworthy, and warrants further investigation. Changes in the tissue morphology, level of hepatocyte degeneration and necrosis, and expression of CYP2E1 correspond well with the observed levels of liver enzymes, kidney parameters, lipid peroxidation and antioxidative enzyme levels.

During inflammation, including sterile inflammation such as that induced by a paracetamol overdose, numerous cytokines are upregulated, with several, most notably TNF-α and IL-1β, implicated as critical mediators of paracetamol-induced hepatotoxicity [69]. In the present study, a histopathological examination performed 24 h after oral paracetamol administration revealed evident centrilobular necrosis, confirming the presence of liver tissue injury. Consistent with these findings, the immunohistochemical analysis revealed focal COX-2 immunoreactivity in paracetamol-treated animals, predominantly localized to centrilobular and perinecrotic regions, whereas minimal or no staining was observed in the control groups. COX-2 staining was predominantly localized to centrilobular regions and areas adjacent to necrotic foci, suggesting the activation of inflammatory signaling pathways in zones of hepatocellular injury.

The observed increase in COX-2 immunoreactivity in paracetamol-treated liver tissue is consistent with previous reports demonstrating upregulation of COX-2 expression in both hepatocytes and non-parenchymal cells in experimental models of paracetamol overdose. Immunohistochemical studies have shown that paracetamol exposure markedly increased COX-2 staining in murine liver tissue compared with controls [69,70]. Moreover, similar elevations in the COX-2 distribution have been reported in rat models of paracetamol-induced hepatotoxicity [71]. Conversely, no COX-2-positive hepatocytes were detected in the Con, Car or CarS groups, which is consistent with the absence of necrotic changes in these animals. In the Car+P and CarS+P groups, COX-2 immunoreactivity appeared more limited and was mainly restricted to focal areas adjacent to zones of necrosis and hepatocyte injury (Figure 3). These descriptive findings are consistent with a possible protective effect of both pure carnosine and the commercial carnosine supplement on local inflammatory activation associated with paracetamol-induced hepatic injury. However, further mechanistic studies are required to corroborate this interpretation, and these results should be considered in the context of other potential interplay between carnosine and paracetamol.

The significance of paracetamol overdose and toxicity becomes evident when observing that it can progress to acute liver injury (ALI) and, if unresolved, ALI may develop into acute liver failure—a condition associated with a high mortality rate. Once ALI is established, there are no specific therapies apart from supportive care, emphasizing the need for novel therapeutic strategies for paracetamol-induced hepatotoxicity and ALI [68]. Recently, liver macrophages have become a major topic of research as potential therapeutic targets in paracetamol toxicity and ALI. The liver harbors the largest population of tissue macrophages, comprising tissue-resident Kupffer cells and bone-marrow-derived monocytes that migrate from the bloodstream into hepatic tissue, giving rise to bone-marrow-derived macrophages. These bone-marrow-derived macrophages account for only a minor proportion of the total hepatic macrophage pool in a healthy liver. However, their proportion can vary under pathological conditions [72].

Both populations of macrophages are Iba1^+^, so we used this marker to assess changes in the macrophage population. Although CD68 is a widely accepted macrophage marker, it is less sensitive for detecting migrating monocytes. Therefore, Iba1 provides more accurate data on the number of monocyte/macrophage cells. Using the ImageJ software, a quantitative analysis of Iba1 immunostaining was performed by determining the number of Iba1-positive cells in liver tissue sections (Table 4).

On the other hand, the number of Iba1^+^ cells was higher in the ConP group compared with the Con group, although the difference did not reach statistical significance. However, it is somewhat unexpected that no statistically significant differences with the Con group were observed. These results are somewhat inconsistent with the findings reported in similar studies. This could be attributed to differences in animal models and the doses applied to induce hepatotoxicity, which were lower compared to similar studies. For example, Jang et al. reported a similar pattern of necrosis and tissue damage, increased hepatotoxicity, and oxidative stress similar to our results. However, the authors reported a significant increase in the macrophage population after paracetamol administration [73]. It should be noted that the paracetamol dose applied to a single animal in their study was almost four times higher than the dose used in ours (400 mg/kg vs. 110 mg/kg, respectively). It is likely that the selected dose represents a sub-hepatotoxic to moderately hepatotoxic regimen in rats, sufficient to induce central necrosis, but limited inflammatory recruitment at 24 h. Higher paracetamol doses or extended time points might produce more pronounced macrophage accumulation.

An apparent discrepancy between COX-2 immunoexpression and the Iba1 findings in the Con and ConP groups was observed. This difference can be explained by the distinct biological and analytical information provided by these markers. COX-2 is an inducible enzyme whose expression reflects inflammatory activation and prostaglandin pathway engagement, and it is frequently upregulated locally in response to sterile necrosis [71]. Conversely, Iba1 primarily reflects macrophage/Kupffer cell abundance rather than their functional activation state. At the 24 h time point and under the applied paracetamol dosing regimen, hepatic injury may preferentially trigger the activation of resident Kupffer cells and localized inflammatory signaling within perinecrotic regions, without a proportional increase in the total number of Iba1-positive cells [70,74]. Furthermore, the focal and spatially heterogeneous nature of paracetamol-induced liver injury may limit the sensitivity of a random field quantitative assessment in the detection of lesion-associated macrophage responses [75,76]. Together, these findings suggest that paracetamol administration at this dose induces a localized inflammatory response characterized by increased COX-2 expression in areas of necrosis, while macrophage accumulation remains limited at the selected time point and under the applied dosing regimen.

The sole administration of carnosine or the carnosine dietary supplement maintained the number of Iba1^+^ cells at a level comparable to the Con group, without statistically significant differences. The number of Iba1^+^ cells in the liver tissue of animals treated with carnosine along with paracetamol (Car+P) showed values similar to those observed in the ConP group. On the other hand, co-administration of the commercial carnosine dietary supplement with paracetamol (CarS+P) was associated with a lower number of Iba1^+^ cells compared with the ConP group, although the difference was not statistically significant.

Despite the presence of typical tissue damage, impaired liver function, and oxidative stress following paracetamol administration, no statistically significant changes in the number of Iba1^+^ cells were detected at the selected time point and under the applied paracetamol dose. In mouse models of paracetamol-induced acute liver injury, the hepatic macrophage population increases over approximately 72 h. However, it is notable that the Kupffer cell numbers initially decline [68]. Considering this, we cannot rule out the possibility that the apparent stagnation of the macrophage population 24 h after paracetamol administration does not reflect the absence of monocyte migration from the peripheral blood, but rather represents a time point in which the influx of monocytes is masked by a decrease in the number of Kupffer cells.

The role of macrophages in the pathogenesis of paracetamol-induced liver injury remains an area of active investigation, with evidence demonstrating heterogeneous and plastic macrophage phenotypes that can exhibit pro-inflammatory or pro-resolving functions depending on microenvironmental cues [77]. This controversy may stem from the heterogeneity and/or plasticity of macrophages, as well as the challenges associated with distinguishing and differentially studying their subpopulations in the liver [78]. Pioneering studies in this field have suggested that, during ALI, circulating monocytes differentiate into pro-inflammatory phenotype of macrophages, whereas the proliferation of the Kupfer cell pool gives rise to macrophages with an anti-inflammatory phenotype. Both populations are morphologically detected by the same markers, like Iba1 [79]. More recent research has suggested that the macrophage ontogeny in ALI is more complex than previously thought. An increasing number of phenotypic subpopulations have been identified, but in general, the current consensus is that, upon liver infiltration, monocytes initially exhibit pro-inflammatory properties, and subsequently begin transitioning toward a pro-resolution phenotype even before the Kupfer cell numbers have fully recovered [68,80]. Since administration of a commercial carnosine dietary supplement (CarS+P) prevents the tissue necrosis observed following a single dose of paracetamol (ConP), the question arises: could carnosine or the commercial carnosine dietary supplement somehow promote a shift in the phenotype of liver macrophages, particularly bone-marrow-derived monocytes, toward an anti-inflammatory and pro-reparative one? While this remains a speculative idea, further studies are needed to investigate the potential immunomodulatory effects of carnosine and its commercial dietary supplement, with particular emphasis on the interaction with relevant macrophage subpopulations.

This study has several limitations that should be acknowledged. Although doses below 200 mg/kg have been employed in published work on paracetamol-induced hepatotoxicity in mice [81,82], producing stable animal models with significant differences in liver function parameters and oxidative stress markers, the more commonly reported dose range is 200–400 mg/kg [83]. In the present study, a dose of 110 mg/kg was applied, according to a previously established model. However, this relatively low dose, in combination with the small sample size, may have been insufficient to produce statistically significant differences between the groups across all the parameters evaluated. The high standard deviations may additionally reflect an inherent biological variability in the small sample, where individual differences in the response to the applied treatment may increase variance estimates. Although the present study was exploratory in nature, and aimed to evaluate the effects of carnosine and a commercial carnosine dietary supplement in paracetamol-induced hepatotoxicity, rather than to compare them with established therapeutic agents, inclusion of a positive control such as N-acetylcysteine may have provided additional context for interpretation of the observed effects. Furthermore, the molecular mechanisms hypothesized to underlie the observed biochemical changes were not investigated in this study, and therefore require further evaluation. Since the inflammatory and macrophage responses in paracetamol-induced liver injury are strongly time-dependent, an analysis at a single 24 h time point represents an important limitation of the present study.

## 4. Conclusions

Carnosine exerted heterogeneous effects in paracetamol-induced hepatotoxicity that depended on the formulation and outcome measures. The pure carnosine pretreatment tended to aggravate biochemical liver injury, whereas the antioxidant-enriched carnosine supplement showed partial hepatoprotective effects, including attenuation of lipid peroxidation, preservation of bilirubin levels, and reduced histological damage. Both formulations decreased the CYP2E1 expression and were associated with less necrosis and COX-2 immunoreactivity compared with paracetamol alone, suggesting modulation of paracetamol bioactivation and inflammatory signaling. However, these tissue-level improvements were not consistently reflected in serum transaminases or antioxidant enzyme activities.

Overall, the effects of carnosine in paracetamol-induced hepatotoxicity appear to vary depending on the formulation used, and do not consistently indicate a uniform hepatoprotective effect. Additional antioxidants present in the commercial dietary supplement likely modified the biological effectiveness of carnosine. Further studies are needed to clarify the conditions determining the protective versus detrimental outcomes of carnosine in paracetamol-induced hepatotoxicity, including an investigation of the underlying mechanistic pathways, exploration of a wider dose range of both paracetamol and carnosine preparations, and inclusion of multiple sacrifice time points to capture the temporal aspects of hepatic injury and recovery.

## Figures and Tables

**Table 1:** Liver function tests according to the animal treatment.

	Con	ConP	Car	Car+P	CarS	CarS+P
ALT (U/L)	58.50 ± 12.52	289.00 ± 390.53	48.13 ± 13.57 ^b^	2905.13 ± 4435.93	80.00 ± 66.56	332.00 ± 338.56
AST (U/L)	240.13 ± 57.32	246.75 ± 110.38	141.38 ± 40.68 ^a^	1877.88 ± 2960.67	238.38 ± 63.17	259.00 ± 59.02 ^c^
Total bilirubin (µmol/)	1.25 ± 0.46	1.63 ± 0.52	1.25 ± 0.71	2.63 ± 1.85	0.88 ± 0.64	1.33 ± 1.03
Direct bilirubin (µmol/L)	0.50 ± 0.17	0.78 ± 0.12	0.54 ± 0.22	1.23 ± 0.93	0.40 ± 0.12 ^b,d^	0.50 ± 0.24

^a^ Significantly different from Con group, *p* < 0.05. ^b^ Significantly different from ConP group, *p* < 0.05. ^c^ Significantly different from Car group, *p* < 0.05. ^d^ Significantly different from Car+P group, *p* < 0.05.

**Table 2:** Kidney function tests according to the animal treatment.

	Con	ConP	Car	Car+P	CarS	CarS+P
Urea (mmol/L)	6.86 ± 0.81	6.46 ± 0.52	6.90 ± 0.78	6.81 ± 1.26	6.69 ± 0.61	6.80 ± 0.82
Creatinine (μmol/L)	17.88 ± 1.73	18.75 ± 1.67	17.63 ± 0.92	17.50 ± 1.85	19.88 ± 1.96	16.83 ± 1.60
Uric acid (μmol/L)	176.00 ± 30.76	155.63 ± 25.65	171.38 ± 28.34	96.00 ± 65.61	173.00 ± 35.08	157.83 ± 24.51

No statistically significant differences were observed among the groups (*p* > 0.05).

**Table 3:** CYP2E1^+^ area of liver tissue.

	Con	Con+P	Car	CarS	Car+P	CarS+P
CYP2E1^+^ (% liver tissue)	12.52 ± 2.53	17.05 ± 3.86 ^a^	11.84 ± 0.36	10.43 ± 3.03 ^b^	11.06 ± 2.04 ^b^	13.18 ± 2.33 ^b^

^a^ Significantly different from Con group, *p* < 0.05. ^b^ Significantly different from ConP group, *p* < 0.05.

**Table 4:** Quantitative analysis of Iba1^+^ cells in liver tissue.

Iba1^+^ Cells	Con	ConP	Car	Car+P	CarS	CarS+P
(*n*)	393.0 ± 94.0	429.1 ± 91.4	358.7 ± 58.0	403.1 ± 112.6	272.4 ± 101.4	334.0 ± 75.0

No statistically significant differences were observed among the groups (*p* > 0.05).

## Data Availability

The original contributions presented in this study are included in the article. Further inquiries can be directed to the corresponding author.
